# Mucormycosis in pediatric oncology patients: a hospital outbreak investigation report

**DOI:** 10.1016/j.infpip.2021.100189

**Published:** 2021-11-20

**Authors:** Ahmed I.H. Saleem, Asim Alsaedi, Maher Alharbi, Shaker Abdullah, Ali Al Rabou, Mona AlDabbagh

**Affiliations:** a Department of Pediatrics, Division of Infectious Disease, King Abdulaziz Medical City (KAMC-Jeddah), Jeddah, Saudi Arabia; b King Abdullah International Medical Research Center, Jeddah, Saudi Arabia; c Ministry of Health, Saudi Arabia; d College of Medicine, King Saud bin Abdulaziz University for Health Sciences (COM, KSAU-HS), Jeddah, Saudi Arabia; e Infection Prevention and Control Department, King Abdulaziz Medical City, Jeddah, Saudi Arabia; f Department of Oncology, King Abdulaziz Medical City, Jeddah, Saudi Arabia

## Introduction

Mucormycosis is an emerging life-threatening fungal infection that occurs as a consequence of environmental exposure. Among stem cell transplant (SCT) recipients, mucormycosis accounts for 8% of invasive fungal infections (IFI), making it the third most common IFI after invasive candidiasis and aspergillosis. [1,2] It was reported as the second most common IFI in Australia. [3] The rate of mucormycosis has increased since the 1980s due to many factors including the increased use of immunosuppressants and the prolonged use of antifungal agents lacking activity against the *Mucorales* species. [4] The rate of mucormycosis in patients with hematological malignancies is highest and especially so among those with acute myelogenous leukemia (AML), with rates ranging between 1-8%. [5,6] Lower rates have been reported in SCT recipients of less than 2%. This rate, however, increases in patients with evidence of graft versus host disease (GvHD). [5,7,8] In addition, patients experiencing iron overload including those on deferoxamine therapy are more prone to disseminated infection. [9] Mucormycosis has been linked to many hospital outbreaks, mainly in neonatology hematology, transplant, surgery, and also in dialysis units. [10] Investigations of these hospital outbreaks revealed that the infections were related to: adhesive bandages, wooden tongue depressors, ostomy bags, water circuitry damage, bed linens, and adjacent building constructions. [8].

Several studies showed that Mucor species were detected in indoor and outdoor environmental air samples in Saudi Arabia. [[11], [12], [13]] Data on the prevalence of mucormycosis in Saudi Arabia is very limited and not well documented. [14] Reports showed that the prevalence rate of mucormycosis in Saudi Arabia is 0.034 cases per 100,000. [15] However, this rate could be an underestimate knowing that the prevalence and incidence rates of diabetes mellitus (DM) and the number of transplants, which are considered major risk factors of mucormycosis, are increasing in Saudi Arabia. [14].

The pediatric hematology/oncology inpatient unit in Princess Norah Oncology Center, King Abdulaziz medical City-Jeddah (KAMC-Jeddah) has 41 beds. It had recently been reconstructed and enhanced to international standards to host immunocompromised pediatric patients. No mucormycosis cases had been seen since 2011 as per unpublished internal data. Here, we present a hospital outbreak investigation of mucormycosis that occurred in this ward during the summer of 2018.

## Materials and methods

### Setting

The Princess Norah Oncology Center at KAMC-Jeddah is a referral center serving the western part of the country. The pediatric oncology/bone marrow transplant units comprise 41 beds in two adjacent wards (wards 11 and 12), with six rooms dedicated to bone marrow transplantation. Both wards are connected by synchronized door systems to regulate airflow. Part of the referral center is a dedicated emergency room for oncologic emergencies, located in ward 10, nearby. The pediatric oncology section accepts nearly 260 referrals and 2,260 visits annually (see Appendix A).

The ward is under a dedicated infection control practitioner who visits the areadaily, provides advice on isolation needs and collects data for related surveillance activities. There is also an assigned environmental health inspector who conducts periodic environmental health assessment rounds, monitors isolation functions and the protective environment rooms, and collects surveillance air samples. The infection control department has seven infection control practitioners and two infection control coordinators reporting to two assigned physicians. Besides, there are two additional sections under the infection control department: the environmental health section staffed with three inspectors, one specialist, and a manager; and the public health section staffed with four nurses and three physicians.

### Outbreak description

**Figure 1:**
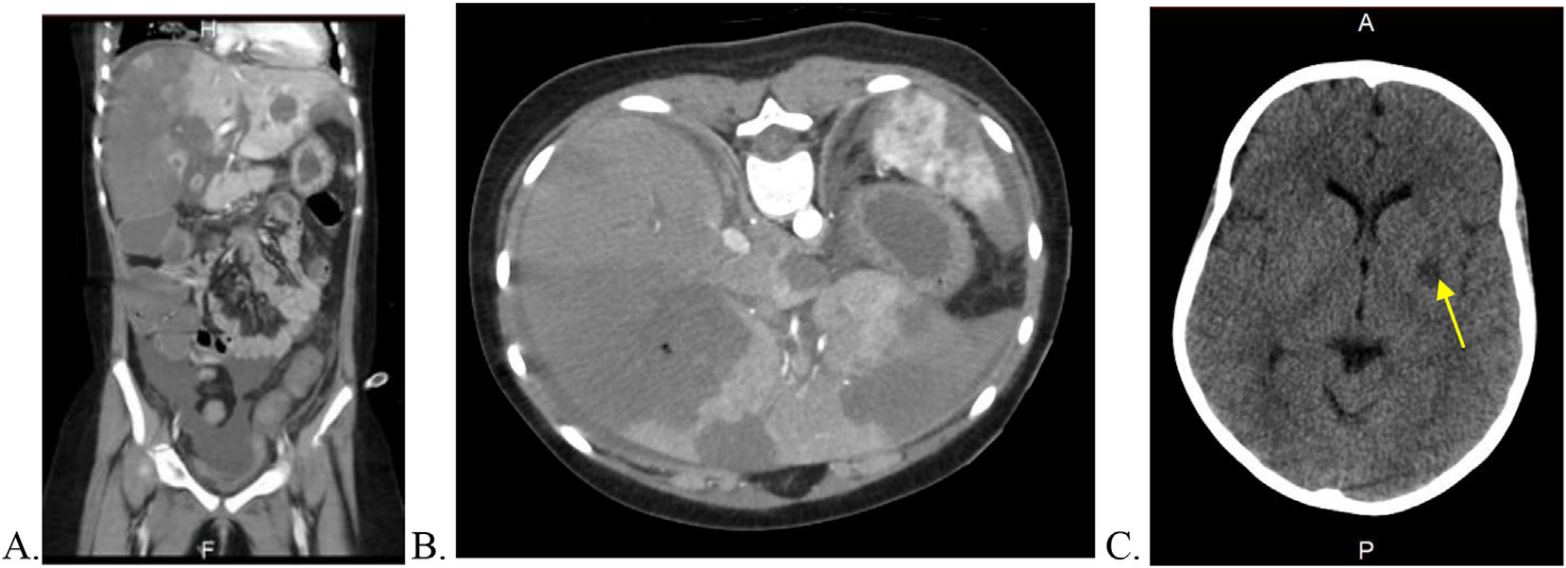
Computed tomography (CT) scan in case 3. A: Coronal section of CT abdomen showing multiorgan infarction involving multiple segments of the liver, the spleen, left kidney, and gall bladder. B: Horizontal section of CT abdomen again showing liver, spleen and kidney infarction. C: CT head with brain infarction in the left corona radiata and lentiform nucleus measuring 1.0 × 1.3cm (Arrow).

**Figure 2:**
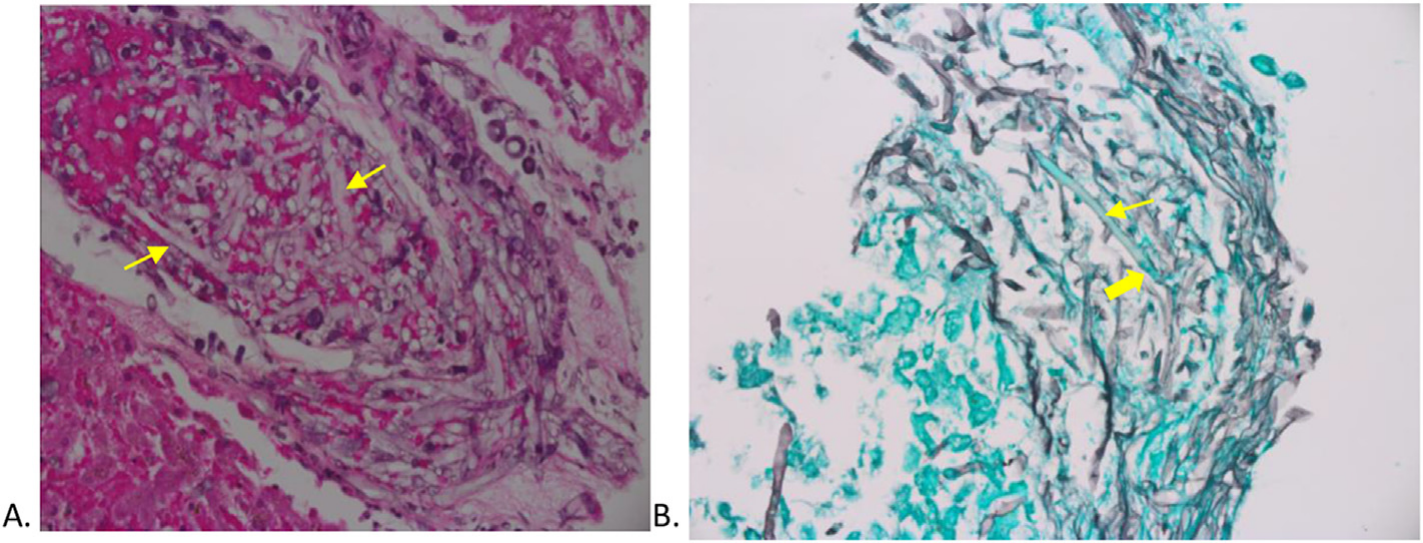
Histopathology of liver biopsy in Case 3. A: H&E staining of the histopathology slide showing extensive necrosis with extensive involvement of branching aseptate fungal hyphae with vascular invasion (arrow head). B: GMS staining clearly demonstrates Mucor spp. with the characteristic broad hyphae branching at 90 degrees angles (thin and thick arrows). H&E: hematoxylin and eosin, GMS: Grocott's methenamine silver stain.

A description of the three suspected/confirmed cases of mucormycosis is summarized in Table I. In the first case (Case 1), the patient was suspected to have mucormycosis due to worsening nodules in the lungs while on caspofungin therapy as seen on a repeat chest computed tomography (CT) scan. Therefore, Amphotericin B lipid complex (Ablecet) was administered. After five days, a black tongue lesion appeared and Posaconazole was then added. She only lived for another 13 days with no histopathological diagnosis. In our index case (Case 2) the patient underwent an operation for bowel perforation previously, for suspected pseudomembranous colitis 16 days after the first case was suspected. The pathology report was consistent with heavy infection with *mucormycosis* Accordingly, the possibility of a mucormycosis outbreak was raised and an outbreak investigation was initiated by the infection control department. In the third case (Case 3), the patient developed typhlitis with rectal gangrene and clinical features of disseminated IFI with multiorgan infarctions 19 days after the first case and only two days after the index case (Figure 1). Her condition quickly deteriorated and she required transfer to the pediatric intensive care unit (PICU). A bedside liver biopsy was obtained, but the patient died soon after. Histopathology results showed extensive necrosis with extensive involvement of branching aseptate fungal hyphae with vascular invasion (Figure 2). Tissue culture subsequently revealed heavy growth of *Mucor* spp. that was identified as a *Mucor* genus without further speciation. A hospital outbreak was then confirmed, and the appropriate corresponding measures were undertaken.

**Table I:** Characteristics of the three confirmed/suspected cases of mucormycosis in a pediatric oncology ward at Nora oncology center, KAMC-Jeddah

	Age (yrs.)	Gender	Underlying disease	Diagnosis	Means of diagnosis	Organs involved	Treatment given	Outcome
Case 1	11	Female	Newly diagnosed ALL with steroid-induced hyperglycemia.	Possible disseminated mucormycosis	Clinical diagnosis based on progression of the disease despite caspofungin therapy. This was further supported after occurrence of a black tongue lesion.	Lung nodules with cavitation, liver, spleen, pancreas, and kidneys dissemination and a black lesion at the base of the tongue	IV Amphotericin B lipid complex, IV Posaconazole was added 4 days later	Death 14 days after clinical suspicion and initiation of treatment
Case 2	12	Female	Newly diagnosed Pre-B cell ALL	Confirmed gastrointestinal/disseminated mucormycosis	Histopathologic examination showed necrotic areas with numerous non-septate broad branching fungal hyphae with vascular and neural invasion consistent with mucormycosis infection.	Gastrointestinal tract with bowel and visceral infarctions	Surgical debridement + IV Liposomal amphotericin B and IV Posaconazole, + Intra-peritoneal irrigation and instillation of conventional amphotericin B	Death 10 days after diagnosis and treatment
Case 3	11	Female	Relapsed AML	Confirmed gastrointestinal/disseminated mucormycosis	Percutaneous liver biopsy showed extensive necrosis with extensive involvement of branching non-septate fungal hyphae with vascular invasion (Figure 2). Tissue culture subsequently revealed heavy growth of *Mucor* spp. (Mucor genus without speciation).	Lungs, and colonic abscesses complicated by rectal gangrene, with multiorgan infarctions including the liver, spleen, both kidneys, gall bladder, and the brain	Amphotericin B lipid complex	Death 1 day after diagnosis and treatment

ALL: Acute Lymphoblastic leukemia; AML: Acute myelogenous leukemia; IV: Intravenous.

### Outbreak investigation

The three cases of invasive mucormycosis were identified within a period of 21 days in August 2018. Therefore, immediate infection control measures were commenced aiming at intensifying measures against fungal infection. These measures included: switching of all patients on any antifungal treatment to amphotericin B or equivalent, starting all high-risk patients on posaconazole prophylaxis, ordering fungal screening for high-risk patients, and obtaining biopsies for all measurable disease detected by imaging studies whenever possible. An active search was undertaken by oncology physicians who were notified by the chairman of the oncology department to submit data of cases of IFI including the submission of data for any clinically-suspected IFI in any pediatric hematology/oncology patient. Furthermore, the microbiology laboratory was contacted to submit any positive microbiological culture for IFI for any pediatric hematology/oncology patient in the preceding three months. In addition, the histopathology department was requested to submit data on all positive IFI histopathology samples for the same patient population during that same timeframe. The pediatric oncology wards were closed; the other inpatients were moved to other areas in the hospital during the three-month period of the outbreak investigation (from September through November 2018).

The outbreak investigation was conducted by the infection control department. The team, led by the director of the infection control department, included one infection control coordinator, one infection control practitioner, the manager of environmental health services, an environmental health inspector, a public health physician, and a public health nurse along with ad hoc members from pediatric infectious diseases section, pediatric hematology oncology section, microbiology department, nursing services, and department of utilities and maintenance. Accordingly, environmental sampling, a review of the medical records of these patients, and a study of potentially implicated patient care activities were performed, to identify common exposures/risk factors for mucormycosis in the three proven/suspected cases.

### Case definitions and finding methodology

Mucormycosis case definition and identification were as follows:

*SUSPECTED CASE OF MUCORMYCOSIS:* Any pediatric hematology/oncology patient admitted after May 1, 2018, who was started on mucormycosis-active antifungal treatment due to clinical and/or radiological suspicion of mucormycosis which developed ≥ 7 days of hospital admission, without any histopathological evidence indicating an alternative diagnosis.

*CONFIRMED CASE OF MUCORMYCOSIS:* Any pediatric hematology/oncology patient admitted after May 1, 2018, who was started on antifungal treatment due to clinical and/or radiological suspicion of mucormycosis that developed ≥ 7 days of hospital admission, with histopathological and/or microbiological evidence of mucormycosis infection.

Patient data were reviewed for all possible cases of IFI over the preceding 3 months before identification of the first mucormycosis case in the pediatric hematology/oncology ward. Clinical, microbiological, and histopathological data were retrieved. Cases were traced back to May 2018; however, no possible cases of mucormycosis were detected during that time.

## Results

An outbreak took place during the summer of 2018 and the cluster of cases was identified in pediatric oncology wards of Princess Norah Oncology center at KAMC-Jeddah. Three suspected/confirmed cases of infection with invasive mucormycosis, who had been diagnosed with hematological malignancies (including one case of AML) were identified within a three-week timeframe (Table 1). The first case was regarded as a suspected case of mucormycosis as there was no histopathological evidence to support the diagnosis, while the two other cases were confirmed by histopathology. All three patients were admitted concurrently to the same ward, received chemotherapy, and experienced neutropenic fever that failed to respond to antibiotic and antifungal therapy. Surgical intervention followed by intra-peritoneal antifungal instillation were performed in one of the cases.

### Tracing of cases

**Figure 3:**
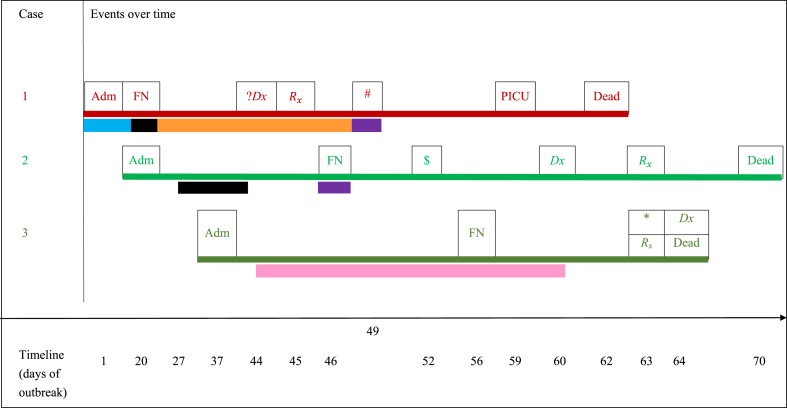
Timeline of events for the three patients. Legend A, events over time: Adm: admission, CT: computed tomography, Dx: Diagnosis confirmed by histopathology, ?Dx: Clinical diagnosis, FN: Febrile neutropenia, PICU: Pediatric intensive care unit, $R_{x}$:Mucormycois-targeted antifungal therapy, #: Black tongue lesion, $: Suspicion of typhlitis, *: Rectal gangrene. DARK RED color denotes CASE 1LIGHT GREEN color denotes CASE 2DARK GREEN color denotes CASE 3. Legend B; significant patient locales in relation to time and other cases throughout the outbreak investigation. EACH SEGMENT COLOR UNDER THE INDIVIDUAL PATIENT TIMELINES REPRESENTS A DIFFERENT ROOM WHERE THE PATIENT WAS PHYSICALLY ADMITTED: LIGHT BLUE color denoting ROOM 11.36. BLACK color denoting ROOM 11.31ORANGE color denoting ROOM 11.23 (index room).  PURPLE color denoting ROOM 11.26PINK color denoting ROOM 11.35.

All common locations shared by these patients were traced. The search showed that in Case 1 the patient had spent the first 20 days of her two-month admission period in room 11.36 (see Appendix A), next to room 11.35 where the patient identified as Case 3 later stayed for most of her in-hospital days (followed by the rapid deterioration and dissemination of fungal infection). However, Case 1 and Case 3 were not present in these adjacent rooms at the same time. Room 11.31 was the common location for Case 1 and Case 2. In Case 1 the patient stayed in room 11.31 for five days but was then moved to room 11.23 (The index room) for the next twenty-three days. Two days after Case 1 left room 11.31, Case 2 was admitted to that same room (i.e 11.31) and remained there for 15 days. Another common room was room 11.26, where the patient in Case 2 stayed for one day before being transferred to the PICU for septic shock that preceded her symptoms of fungal disease. Three days after Case 2 left room 11.26, Case 1 was moved there for one day, and that was concurrent with the development of worsening symptoms that later progressed to disseminated mucormycosis. Both patients died on the same PICU bed. Figure 3 outlines the overlapping timelines between all three cases including the time of admission, onset of febrile neutropenia, mucormycosis clinical or pathological diagnosis, the corresponding initiation of treatment, and death.

### Review of potentially implicated factors

An exhaustive review of potentially implicated patient care activities was completed, including all interventions and radiological investigations. A food and water safety questionnaire (see Appendix B) were generated to collect data from parents of the included patients about the type and sources of food and water during hospitalization. Inspection rounds were conducted at multiple patient care areas to identify any possible source of contamination, those areas included: the two hematology/oncology wards, surrounding patio, housekeeping areas, laundry, medication preparation rooms, kitchen, angiography suite, and the PICU. A study of air parameters including: pressure differentiation, temperature, humidity, and the adequacy of high-efficiency particulate air (HEPA) filters were performed. This was done in addition to the review of periodic air sampling reports and recent maintenance activities at the pediatric hematology/oncology wards. Following the inspection of the patients' rooms, dust accumulation was observed on the return air grills in some rooms, mainly room 11.23, which tested positive for *Mucor* spp. It is important to mention that beside the five colony forming units (CFU) of Mucor spp. isolated from air exhaust vent diffuser in room 11.23, random environmental samples from different surfaces were collected in both wards 11 and 12. Samples from adjacent rooms revealed growth of Aspergillus spp, Penicillium spp, and other non-specified molds in samples collected from air, toilet tiles, oxygen sockets and food trolleys which indicated dust contamination and defective air filtration that is below standards for such a protective environment.

### Interventions for infection control

The possibility of air contamination secondary to malfunctioning heating, ventilation, and air conditioning (HVAC) system was raised and thus immediate evacuation of all patients in pediatric hematology/oncology wards was recommended until the completion of all corrective measures. Corrective measures of the HVAC system included replacement of all HEPA filters in the wards, cleaning and disinfection of supply and return air grills, and adjustment of temperature and relative humidity to be within 21^0^C–24^0^C and a maximum of. 60%, respectively in order to meet the American Society for Heating, Refrigeration, and Air-Conditions Engineers (ASHRAE) standards. Once the HVAC system repair and adjustment were completed, the area was kept under close observation and monitoring for four weeks for any evidence of recurrence of dust accumulation or fungi and was then cleared by the infection control department. After the re-opening of the wards, the area was ready to receive both active in-patients that were moved to other hospital areas during the outbreak investigation, as well as new patient admissions. A simultaneous process of continuing close observation and monitoring was implemented to prevent the recurrence of such incidence in the future. No new cases of IFI were detected in the following six months.

## Discussion

*Mucorales* belong to the *Mucormycotina* subphylum that is further subclassified into four main genera that cause mucormycosis, *Rhizopus, Mucor, Rhizomucor* and *Lichitheimia*. [16,17] They grow rapidly in-vitro with colonies that cover the agar surfaces and expand in height displaying a fluffy appearance. Microscopically they are non-septate, or pauci-septate hyaline broad hyphae. They branch irregularly and at right angles with high predilection for angioinvasion, thrombosis, and tissue necrosis and abscess formation. [18,19].

IFI with *Mucorales* carry a high mortality rate, according to the host and site of infection, generally ranging from 35% in patients with no underlying conditions up to 66% in patients with malignancies. Some reports revealed that disseminated and gastrointestinal mucormycosis carry the highest mortality rates of 85% and 96% respectively. [8] Few antifungal susceptibility studies are available in the literature. It is indicated that amphotericin B displays the lowest minimum inhibitory concentration for *Mucor* spp. as compared to other antifungal agents, making it the drug of choice, followed by Posaconazole. [20].

Here we report an outbreak of mucormycosis involving three pediatric oncology patients during their hospitalization in the summer of 2018. The two patients with confirmed cases primarily developed mucormycosis of the gastrointestinal tract while the suspected case developed primarily lung disease with dissemination and a tongue lesion afterwards. Infection control investigations confirmed the presence of *Mucor* spp. on the surface of the return air grills in some rooms inside the wards, mainly room 11.23, where these patients were admitted. This suggests airborne transmission of the infection, although the mechanism of gastrointestinal involvement is not fully explained.

Mucormycosis is a rare lethal infectious disease that mainly affects the immune compromised population. [9] Oncology patients, and in particular those with hematologic malignancies have the greatest risk for invasive mucormycosis infection. [9] Developments in modern medicine and recent advances in cancer chemotherapeutic management as well as the larger scale of organ transplantation together prolonged the survival of such immunocompromised patients. This has brought about a rise in the rates of invasive mucormycosis since the 1980s. [3,9] The increasing use of immunosuppressive drugs, and the prolonged administration of antifungal agents that lack activity against Mucorales such as voriconazole, are well known risk factors for severe mucormycosis. [9,21] Voriconazole does in fact enhance *Rhizopus oryzae* virulence. [10] Besides, the epidemiology of mucormycosis differs according to the geographic location, age group, and population. In the Indian subcontinent for example, rhino-cerebral and cutaneous disease are reported more commonly than pulmonary and gastrointestinal mucormycosis in contrast to western countries where pulmonary and gastrointestinal diseases are more prevalent. [[22], [23], [24], [25]] The literature has few publications on mucormycosis outbreak investigation, especially in the pediatric age group. More reporting of such outbreaks provides better opportunities to study this emerging infectious disease among the immune-compromised population. Thus, more data can be made available to design preventive measures against such a devastating infection. [26].

In fungal outbreaks, it is not easy to identify if it is nosocomial or community acquired. [27] The main difficulty lies with finding the common source of nosocomial infection. [28] Also, the poor yield of environmental sampling makes the process of outbreak tracing more difficult. [29] Another issue is the possibility of a pseudo-outbreak, where common molds in the environment can be recovered from environmental samples during investigation further complicating the tracking process. [30] According to the anatomic location of the infection, routes of transmission differ, whether by inhalation of the spores as in pulmonary and rhino-orbital infections, or by way of ingestion of foods contaminated with the spores as in gastrointestinal and soft tissue infections. [30,31] Environmental checks and sampling of all possible patients' commonalities is important. Air sampling can be of great value to trace a possible source of the outbreak in addition to checking air ventilatory systems and water leaks or faulty constructions, [28] to help identify airborne fungal spores. [32] Aging facilities pose additional challenges against the implementation of the new recommendations regarding air handling or other infection control measures.^33^ Fungal outbreaks are also commonly related to hospital construction,^34^ therefore, before opening new inpatient wards (especially those for cancer and transplant patients), it is mandatory to carry out frequent air and environmental samplings along with strict serial decontamination measures. An outbreak investigation in a pediatric oncology ward in the UK reported by Garner *et al.*, 2008, indicated that a defect in wall plastering was found in one of the patient toilets. [32] Hospital outbreaks have also been linked to contaminated tapes, medical bandages, tongue depressors, ostomy bags, and in prepared medications in ICU patients. [8] A study by Llata *et al.* on mucormycosis outbreak investigation emphasized the need to quantify the burden of this emerging disease in the United States, as this may not only affect the immunocompromised.^33^ The authors also pointed out that airborne fungal spores could be anywhere in the hospital or outside, and that in-hospital transfer is also a risk.^33^ Interestingly, mucormycosis is associated with seasonal variation, with higher rates in summer and autumn. This is because the prevalence of fungal spores in nature is affected by season. [29].

The major limitations of this report include the lack of diagnostic confirmation for Case 1; thus, her diagnosis was only based on clinical suspicion. Earlier confirmation in this case could have led to earlier initiation of infection prevention measures and probably prevent further cases from occurring. Also, for the other two cases, we could not send the isolated organism for PCR/gene sequencing, nor did we have phylogenotyping for the *Mucor* spp. This might have helped identify the source of infection in outbreak tracing. In addition, although no further cases of mucormycosis have been reported in our center since the time of this outbreak, we cannot confidently say that the disease was not missed in other oncology cases that died, since we do not perform autopsy on patients in Saudi Arabia other than by court order for selected forensic cases.

## Conclusion

Invasive mucormycosis is an ominous, emerging life-threatening infection especially among the immunocompromised. Prompt aggressive infection control measures are vital, with special focus on air sampling and environmental survey. This investigation highlights the importance of the role of operational management and maintenance and engineering departments, inoutbreak management, in assuring the proper functioning of HEPA filters, laminar airflow, and optimal pressure and humidity. In our study we concluded that dust accumulation reflected air contamination and failures in the air filtration process. This indicated system failure in air quality monitoring in high-risk areas, and this was corrected following engineering control and corrective actions. Regardless of the ease with which investigation can be directed towards a certain source (by ingestion or airborne inhalation), it is essential to perform meticulous and exhaustive investigations towards all possible causes and routes. Therefore, collaborative measures are crucial, and appropriate proactive measures should be taken as soon as possible given the critical time factor. In addition, the importance of cleaning to prevent IFI cannot be over-emphasized. One year since that incident, no further cases of mucormycosis were reported in our institution.

## Ethical considerations

Approval of the institutional review board was secured prior to submission for publication.

## Credit author statement

Ahmed Saleem: Collected the date and wrote most of the manuscript.

Maher Alharbi: Was involved in the infection control management of the cases, contributed to the writing of many parts of the infection control part of the manuscript.

Shaker Abdullah: Was involved in the medical management of the cases, contributed to the study design and writing.

Asim Alsaedi: Was the chairman of infection control department who led the infection control management of the cases, contributed to the writing of many parts of the infection control part of the manuscript.

Ali Al Rabou: Was involved in the infection control investigation. He wrote most of the queries to the infection control issues in the revised manuscript and wrote down the data on Muco in KSA.

Mona AlDabbagh: The Infectious disease doctor who identified the patients and initiated the outbreak investigation. Was the principal investigator of the study with leading role in the design, conduct, writing and editing of the manuscript.
