# Supplementary material for: Mucormycosis in pediatric oncology patients: a hospital outbreak investigation report
Source: Infect Prev Pract. 2021 Nov 20;3(4):100189. doi: 10.1016/j.infpip.2021.100189 (PMC8696282; doi:10.1016/j.infpip.2021.100189)
Supplement: Multimedia component 1 [file mmc1.docx]

**Appendices**

**Appendix A: Oncology wards layout/map**

**Appendix B: Food and water safety questionnaire**

**Appendix A:**

***Layout of both oncology wards 11 and 12 interconnected by synchronized doors.***

**
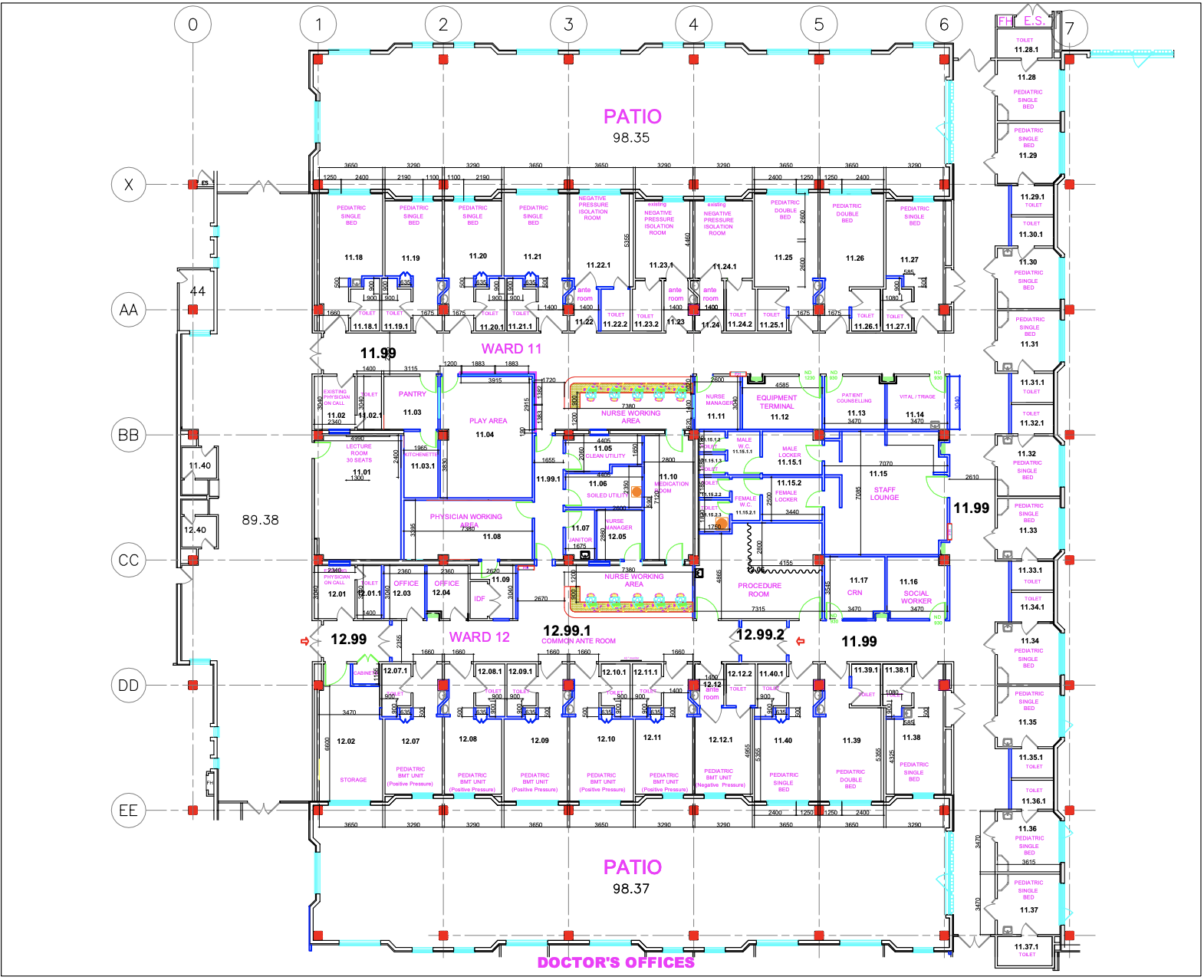
**

***The room indicated by the red square shows where Mucor spp. were isolated. Rooms indicated in orange squares are the other rooms used by the affected cases in the series. Rooms 11.26 and 11.31 were occupied by both cases #1 and #2 in alternative times.***

**Appendix B:**

***Food/water Safety Survey:***

Patient MRN # -----------------------

1- Child ‘s food prepared by

🞏Mother 🞏Father 🞏Child sitter 🞏Other ……

2- Are you aware of food/water contamination routes?

🞏Flies 🞏Dirty hands 🞏Dust 🞏Filthy surroundings 🞏Toilet and urinary 🞏No idea/nothing

3- Length of time raw foods are stored prior to preparation at home is

🞏One day 🞏Between 1 day and 6 days 🞏Between 1 week and 3 weeks 🞏 One month and above

4- Places that raw foods are stored

🞏Fridge 🞏Somewhere in kitchen 🞏Outside yard 🞏Should not be stored 🞏Other ……

5- The use of substances/chemical for raw food cleaning?

🞏Yes 🞏No

If yes, what is the substances/chemical used for cleaning raw food

🞏Salt 🞏Vinegar 🞏Vegetable cleanser solution 🞏Other……

6- Frequency of hand washing when cooking/preparing food

🞏Before cooking/preparing food

🞏Before and after cooking/preparing food 🞏Before, after and during preparing food 🞏More than three times 🞏Don’t remember

7- When cooking utensils/milk bottles are usually washed?

🞏Before preparing food 🞏After preparing food 🞏Before and after serving the food

8- How cooking utensils/milk bottles are washed?

🞏Soap and water 🞏Only water 🞏Heating 🞏Other……

9- List the sources you are using for water consumption (drinking, cooking etc.)

🞏Tap water 🞏Bottled water 🞏Other…...

10- Do you use water filter at home?

🞏Yes 🞏No

11- Apron was used when preparing and serving food to the child at home?

🞏Yes 🞏No 🞏Sometimes 🞏Not sure

12- What do you use tap water for?

🞏Cooking food 🞏Washing vegetables and fruits 🞏Washing plates and utensils 🞏Hand washing 🞏Regular drinking, making coffee and tea…etc. 🞏Personal hygiene (bating, brushing teeth...etc. 🞏Washing items and cloths 🞏Other…………

13- Have you consumed hospital tap water for the last 60 days (coffee, tea, drinking…etc.)?

🞏Yes 🞏No 🞏Not sure

14- Over the last month, most of the child’s food was from

🞏 home 🞏 hospital 🞏venders outside

15- Last time that the child suffered from diarrhea and abdominal cramp after ingestion of food /water was on

🞏Last admission 🞏Last month 🞏Last 6 month 🞏Last year 🞏Cannot remember 🞏Never suffer 🞏Other…………
